# PROVIDING DEMENTIA CARE ACROSS LOW-RESOURCE COMMUNITIES

**DOI:** 10.1093/geroni/igad104.1897

**Published:** 2023-12-21

**Authors:** Sarah Holmes, Kirsten Corazzini

## Abstract

The growing number of persons living with dementia (PLWD) require ongoing care and supportive services, but dementia care access and quality are more limited in communities with fewer health resources and these limitations are exacerbated by current dementia care workforce shortages. Across low-resource communities—including poorer urban and rural settings—health resources tend to be limited and PLWD are predominantly socioeconomically disadvantaged and have few personal financial resources to contribute to care provision. Although there is growing evidence about the consequences of care inequities for PLWD, less is known about key issues and opportunities for improving dementia care in low-resource communities. This symposium features presentations that highlight unique social and economic challenges and opportunities for providing dementia care in low-resource communities. The first presenter reports results from a community-based, participatory research study in four long-term care settings in federally designated medically underserved areas (two rural, two urban) that highlights strategies used by long-term care providers to recruit and retain dementia care staff and describes their perspectives on quality dementia care. The second presenter shares findings from an innovative pilot program of case management services to address challenges of PLWD who are living alone in the community through collaboration with local paramedicine services. The third presenter provides an overview of the Person-Centered Options Counseling Certification program with a discussion on strategies for implementing person-centered dementia care initiatives in low-resource communities. This symposium reveals complexities in providing care for PLWD in diverse low-resource communities and generates directions for addressing dementia care inequities.

